# Hyaluronan in liver fibrosis: basic mechanisms, clinical implications, and therapeutic targets

**DOI:** 10.1097/HC9.0000000000000083

**Published:** 2023-03-17

**Authors:** Jieun Kim, Ekihiro Seki

**Affiliations:** 1 Karsh Division of Gastroenterology and Hepatology, Department of Medicine, Cedars-Sinai Medical Center, Los Angeles, California, USA; 2 Department of Biomedical Sciences, Cedars-Sinai Medical Center, Los Angeles, California, USA

## Abstract

Hyaluronan (HA), also known as hyaluronic acid, is a glycosaminoglycan that is a critical component of the extracellular matrix (ECM). Production and deposition of ECM is a wound-healing response that occurs during chronic liver disease, such as cirrhosis. ECM production is a sign of the disease progression of fibrosis. Indeed, the accumulation of HA in the liver and elevated serum HA levels are used as biomarkers of cirrhosis. However, recent studies also suggest that the ECM, and HA in particular, as a functional signaling molecule, facilitates disease progression and regulation. The systemic and local levels of HA are regulated by *de novo* synthesis, cleavage, endocytosis, and degradation of HA, and the molecular mass of HA influences its pathophysiological effects. However, the regulatory mechanisms of HA synthesis and catabolism and the functional role of HA are still poorly understood in liver fibrosis. This review summarizes the role of HA in liver fibrosis at molecular levels as well as its clinical implications and discusses the potential therapeutic uses of targeting HA in liver fibrosis.

## INTRODUCTION

The extracellular matrix (ECM) maintains physical tissue and organ architecture under physiological conditions. Elevated production and accumulation of ECM in liver tissues are closely associated with the progression of fibrosis, and the concentrations of ECM in liver tissues and blood serve as biomarkers of fibrotic diseases, such as cirrhosis. The ECM plays a more complex role in regulating cellular biology and behavior through the activation of receptor-dependent intracellular signaling.^1^,^2^ The 2 major molecular classes of ECM are fibrous proteins (eg, collagen, fibronectin, elastin, and laminin) and glycosaminoglycans [eg, hyaluronan (HA) and heparan sulfate]. Among glycosaminoglycans, HA is a highly anionic and unbranched molecule without sulfate and core protein. HA is commonly known as hyaluronic acid in hepatology clinics and is used as a biomarker that is elevated in the blood of patients with cirrhosis.^1^,^3^ Until recently, it was unclear whether HA is a bystander component of deposited ECM or a biologically active component. Unlike the lung fibrosis and cancer research fields, the molecular mechanisms of HA-mediated disease progression of liver fibrosis are poorly understood.^3^,^4^ In this review, we summarize the current knowledge of HA biogenesis and catabolism, regulatory mechanisms, molecular size-dependent functions, clinical implications, and the therapeutic potential of targeting HA in liver fibrosis.

### HA as a biomarker for liver fibrosis

HA is used as a noninvasive biomarker of liver fibrosis. Elevated serum levels of HA correlate well with disease progression in liver fibrosis and cirrhosis. Moreover, fibrosis scores, including the enhanced liver fibrosis score, HepaScore, and Fibrospect II, utilize HA in their algorithms.^5^ Serum HA levels are higher in HCV patients with cirrhosis compared with patients without cirrhosis and are even higher in HCV patients with HCC compared with HCV patients with cirrhosis.^6^ HCV patients who achieve sustained response after treatment have reduced serum HA levels.^7^ Similarly, serum HA levels in HBV patients with fibrosis are high, whereas serum HA levels after anti-viral therapies are low.^8^–^11^ In NAFLD and alcohol-associated liver disease (ALD) patients, serum HA can differentiate between patients with and without cirrhosis. These reports indicate that noninvasive measures of serum HA are useful for the diagnosis of cirrhosis and as an indicator of fibrosis resolution after treatment, independent of the etiology of liver fibrosis. However, some reports suggest the changes in the HA levels slightly vary depending on their etiologies. The sensitivity and specificity of HA are high for HBV-associated fibrosis (90.9% and 98.1%, respectively),^12^ but they are moderate for ALD-mediated fibrosis (82.8% and 69%, respectively).^13^ Also, serum HA levels are high in alcohol-associated cirrhosis when compared with those in NAFLD cirrhosis.^14^ When compared with other biomarkers, the sensitivity of HA alone is greater than type 4 collagen 7S (T4C7S), N-terminal propeptide of type III collagen (PIIINP), TIMP1, laminin, but not M2BPGi, Pro-C3, FIB-4, APRI, and enhanced liver fibrosis score; the specificity is higher than T4C7S, laminin, M2BPGi, Pro-C3, FIB-4, APRI, enhanced liver fibrosis score, but not PIIINP and TIMP1 in HBV fibrosis patients.^9^,^15^,^16^ In ALD, the studies reported HA, as a single marker, is better than PIIINP, APRI, and FIB-4 score.^14^,^17^ However, to diagnose early-stage fibrosis in NAFLD, HA is not as good as type 4 collagen and T4C7S.^18^ Serum HA levels are good for differentiating between F2 and F3 but not sensitive to differentiating F1 and F2 fibrosis or diagnosing mild liver fibrosis.^15^,^19^ The limitations of HA alone may be overcome by the combination of HA with other noninvasive biomarkers (eg, PIIINP, Pro-C3, FIB-4) and imaging systems (eg, magnetic resonance elastography, Fibroscan). HA may complement current screening tests for suspected liver disease patients. For example, HA can identify asymptomatic HCV-infected patients from blood donors.^20^ The combination of HA with cytokeratin-18 M30 improves to differentiate NAFLD patients with fibrosis from those without fibrosis.^21^ Addition of HA to the currently available fibrosis scores to improve the prediction and diagnostic accuracy of cirrhosis and HCC is also of special interest. Although FIB-4, one of the current fibrosis scores, predicts future HCC development,^22^ it is not as accurate in patients with type 2 diabetes compared with nondiabetic patients. A combination of FIB-4 and HA during risk stratification for incident cirrhosis and HCC reduces the false-positive rate without increasing the false-negative rate in diabetes patients.^23^ Thus, the addition of HA to the current diagnostic system may improve the prediction and diagnostic accuracy of liver disease, contributing to the reduction of the liver disease burden in the future.

### Serum HA levels are regulated by LSECs

**FIGURE 1:**
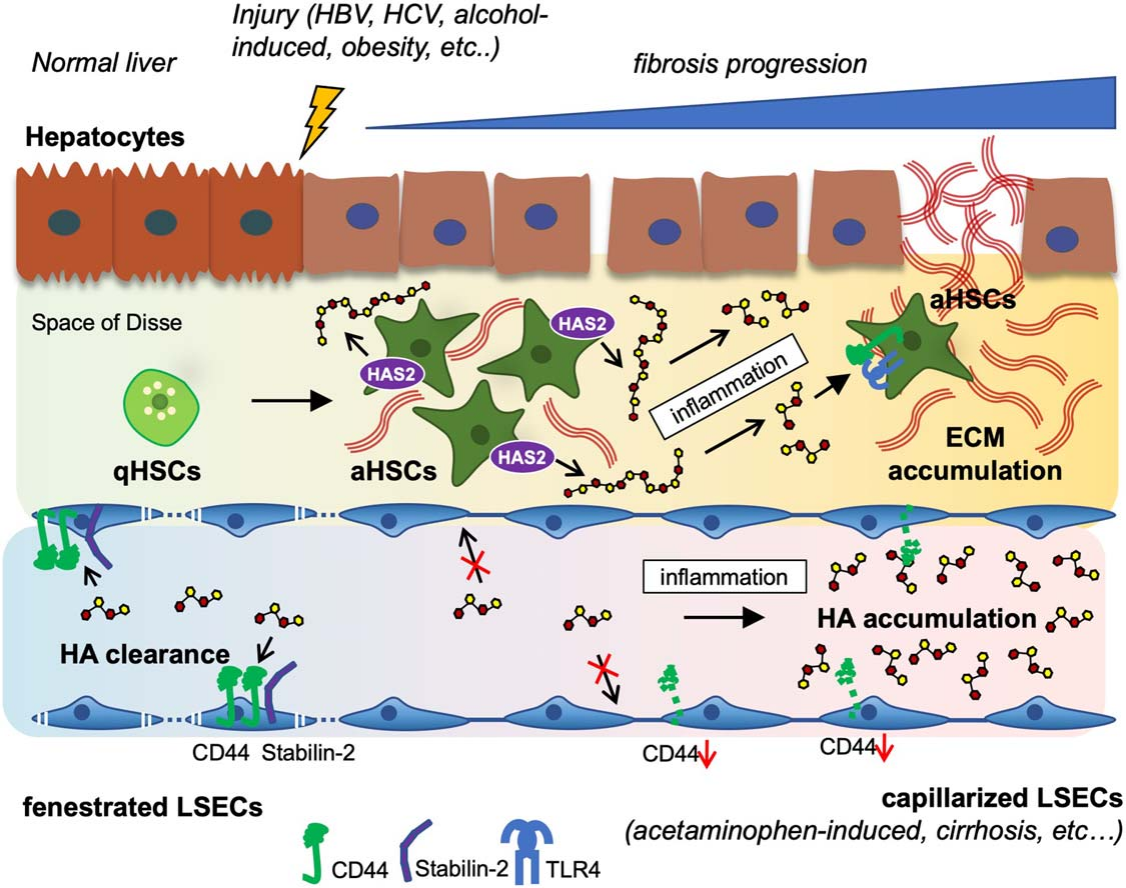
Schematic illustration of the dynamic role of hyaluronan (HA) during the progression of liver disease. When the liver is healthy, low molecular mass form of hyaluronic acid (LMM-HA) are found in circulation. Fenestrated LSECs are responsible for HA clearance by eliminating circulating HA through CD44 and Stabilin-2. Quiescent HSCs (qHSCs) hardly express hyaluronan synthase (HAS2) responsible for HA production in the liver. After the liver injury, the serum and hepatic HA level is increased by LSEC dysfunction and HA hypersynthesis. In acetaminophen-induced liver injury or cirrhosis, LSEC clearance of HA is disrupted with LSEC capillarization. As a result, circulating HA accumulates in the blood. In the injured liver of hepatitis B and C, alcoholic liver disease, or non-alcoholic fatty liver disease, qHSCs transdifferentiate into activated HSCs (aHSCs) that upregulate HAS2 and produce HMM-HA. These highly proliferative aHSCs overexpress HAS2 and HA, leading to HA accumulation. In the presence of inflammation, a major hallmark of liver disease progression, HMM-HA is converted to a LMM-HA. This profibrogenic LMM-HA promotes HSC activation and extracellular matrix (ECM) production through CD44 and TLR4, resulting in liver fibrosis.

HA is constantly produced and is present in almost all tissues, including the skin, surrounding blood vessels, lung bronchioles, and circulation.^24^ The half-life of HA is only 2–5 minutes, highlighting the rapid turnover of circulating HA. One-third of circulating HA is replaced daily. LSECs are responsible for the uptake, degradation, and elimination of 90% of HA.^25^–^28^ LSECs express CD44 and Stabilin-2, also known as the HA receptor for endocytosis. These receptors are the major clearance receptors for circulating HA. Under physiological conditions, HA contents are low in circulation (<50 ng/mL) and very low (~1.5–2 μg/g) in normal liver tissues.^28^ In contrast, serum HA contents are increased to >100 ng/mL in patients with liver fibrosis or cirrhosis (≥F3).^4^,^15^ One of the mechanisms responsible for elevated serum HA levels in patients with cirrhosis stems from the inability of LSECs to eliminate circulating HA. LSEC elimination of HA is disrupted in cirrhosis, which may involve the “capillarization” of LSECs and the downregulation of CD44 in LSECs, preventing HA endocytosis^29^ (Figure 1). As a result, circulating HA accumulates in cirrhosis. Acetaminophen-induced acute liver injury causes LSEC dysfunction and elevation of circulating HA.^30^,^31^ Based on these findings, serum HA levels are used as a marker of LSEC dysfunction.^29^ Because the HA clearance-LSEC dysfunction hypothesis is reasonable, other mechanisms, such as hypersynthesis of HA, have not been carefully considered in the liver fibrosis field for a long time.

### HA is actively synthesized during liver fibrosis progression

**FIGURE 2:**
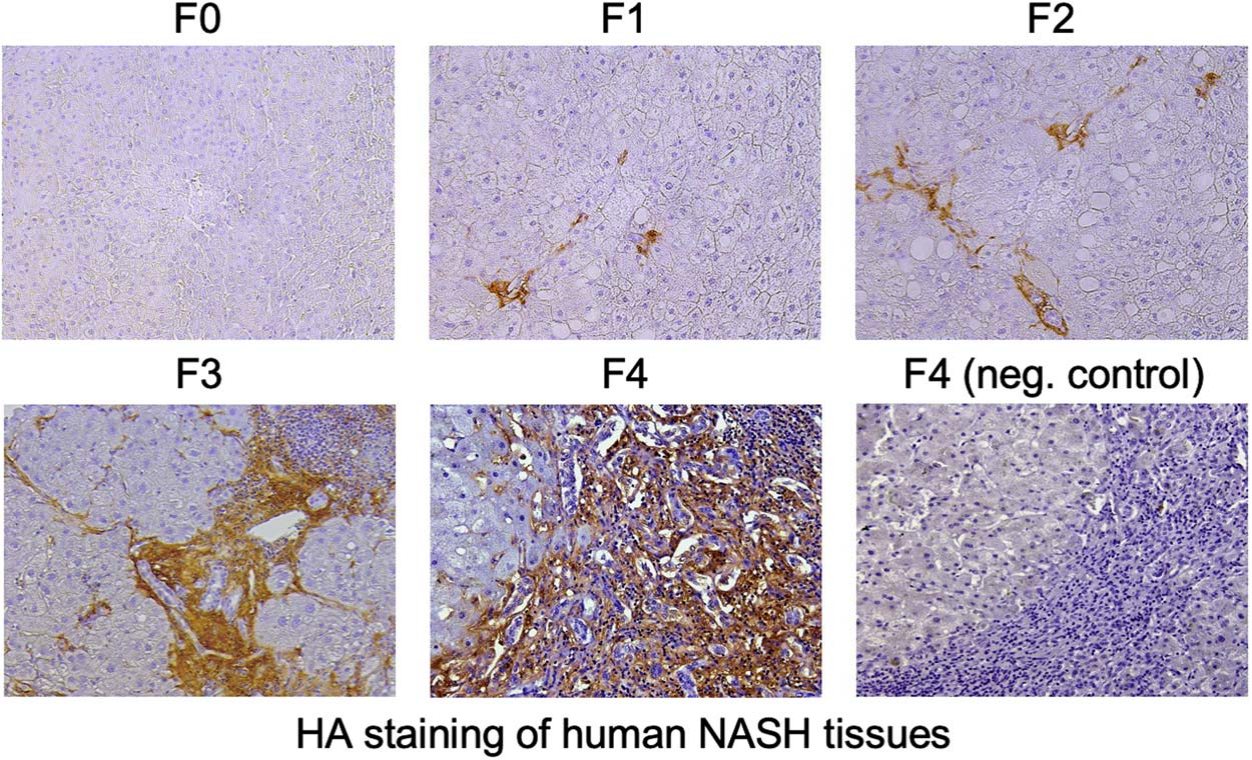
Staining for hyaluronan (HA) in human NASH tissues. HA staining in liver sections of patients with NASH-mediated liver fibrosis (F0-F4). HA-binding protein (HABP) was used for staining HA. Negative controls are stained with HABP following treatment of the tissue sections with 1 mg/mL *Streptomyces hyalurolyticus* hyaluronidase for 1 hour at 37 °C.

**FIGURE 3:**
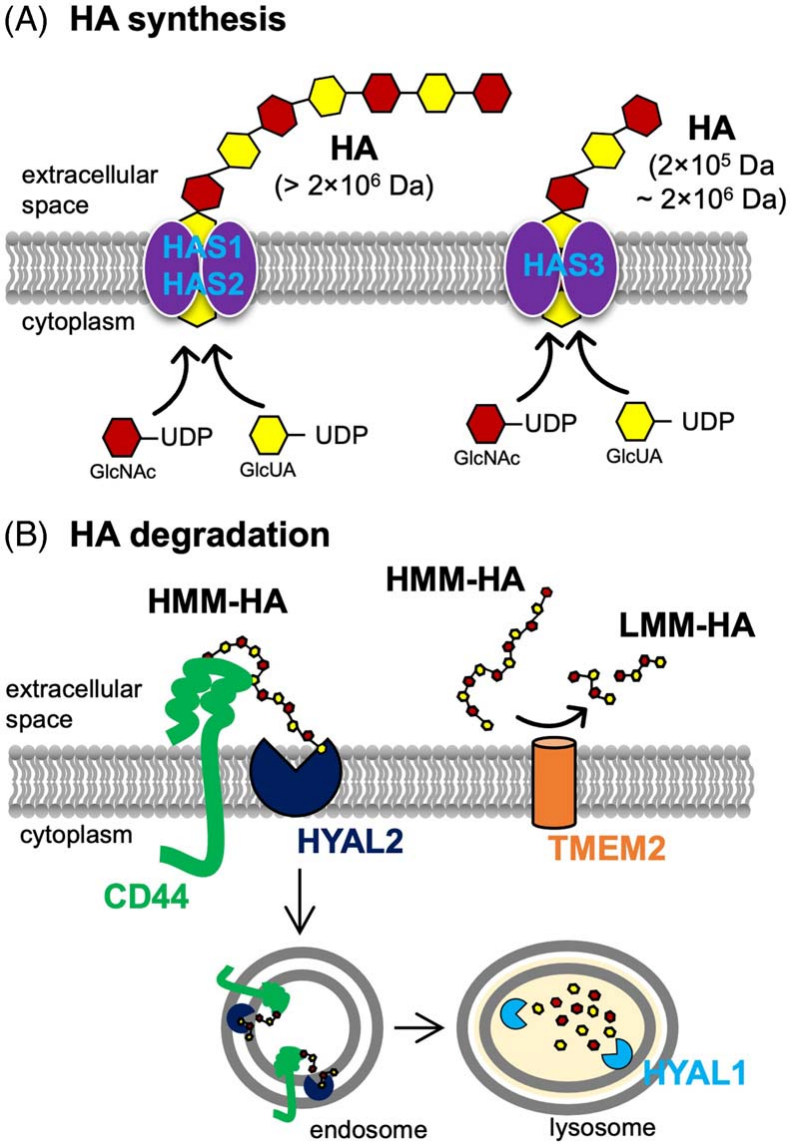
Mechanism of hyaluronan (HA) synthesis and degradation. (A) Mechanism of synthesis of hyaluronan (HA) by hyaluronan synthase (HAS). HA biosynthesis is catalyzed by HAS at the inner surface of the plasma membrane. HAS uses 2 cytosolic substrates, UDP-glucuronic acid (UDP-GlcUA) and UDP-N-acetyl-glucosamine (UDP-GlcNAc), and extrudes the growing polymer through the membrane to form elongated HA. The HAS family includes three isoforms (HAS1, HAS2, and HAS3) that produce different molecular mass HA. HAS1 and HAS2 synthesize high molecular mass (HMM) HA (>2×10^6^ Da), and HAS3 generates slightly lower molecular mass HA (2×10^5^–2×10^6^ Da). (B) The endolytic mechanism of degradation of HMM-HA (1×10^6^–10^7^ Da) by hyaluronidase (HYAL). HYAL1 and HYAL2 are the major HYALs that degrade HA. The process of HA cleavage is initiated by HYAL2, a membrane-anchored protein that acts in cooperation with CD44. HYAL2 degrades HMM-HA into fragments of approximately 1–2×10^4^ Da. These fragments are then internalized into endosomes and further cleaved to oligosaccharides by HYAL1 in lysosomes. Transmembrane protein 2 (TMEM2), a type II transmembrane protein with intrinsic HYAL activity at neutral PH, acts as a cell-surface HYAL to break down HMM-HA into low molecular mass (LMM)-HA.

HA is composed of repeating disaccharides D-glucuronate and N-acetyl-D-glucosamine, indicating that HA is a sugar-based molecule, not a protein. There is no direct antibody to detect HA in tissue staining, so HA-binding protein is commonly used as a surrogate. Based on HA-binding protein staining (Figure 2), HA is almost undetectable in the healthy or uninjured liver (F0). HA accumulation began in early fibrosis (F1–2) and dramatically increased in F3–F4 fibrosis (Figure 2). HA deposition is observed mainly in fibrous bands but not in the liver parenchyma. HSCs are the precursors of ECM-producing myofibroblasts in liver fibrosis and the main cellular source of HA in the liver.^4^,^32^ HA is synthesized by a membrane-bound enzyme, HA synthase (HAS).^3^ HAS synthesizes HA using uridine diphosphate (UDP)-α-D-glucuronate (UDP-GlcUA) and UDP-α-N-acetyl-D-glucosamine (UDP-GlcUAc) as substrates (Figure 3A). HAS has 3 isozymes: HAS1, HAS2, and HAS3.^33^ Of the 3 HAS enzymes, HAS2 is the most powerful and critical. Global knockout of HAS2 in mice is embryonic lethal due to failed cardiac development, whereas HAS1 and HAS3 knockout mice are viable.^34^–^36^ Both HAS1 and HAS2 synthesize high molecular mass (HMM) HA (>2×10^6^ Da); however, the enzymatic activity of HAS1 is not as strong. In contrast, HAS3 synthesizes lower molecular mass HA (2×10^5^–2×10^6^ Da).^33^ These isozymes synthesize HA differently depending on cytoplasmic sugar availability. HAS1 is inactive without a UDP-sugar supply, but HAS3 does not depend on UDP-sugar levels. HAS2 activity increases with UDP-sugars content.^37^ This suggests that HAS1 may significantly affect HA production with increased sugar availability in diabetes and obesity.

HAS3 knockout mice develop liver fibrosis similar to wild-type mice,^38^ suggesting that HAS1 or HAS2 is more likely to regulate liver fibrosis. In the normal liver, levels of the HAS enzymes and HA are very low. Upon HSC activation, HAS2 is the most inducible isozyme, and its expression is dramatically elevated, facilitating HA synthesis (Figure 1). In contrast, HAS1 and HAS3 upregulation after HSC activation are not as dramatic.^4^ HSCs are responsible for HAS2 expression and HA production in liver fibrosis in a study of mice lacking HAS2 expression in their HSCs.^4^ Mice lacking HAS2 expression in their HSCs exhibit reduced HSC activation and fibrosis, along with abolished HA deposition in the liver and no upregulation of circulating HA compared with baseline. An *in vivo* unbiased targeted genomic screening strategy demonstrates HAS2 as one of the 5 most important and novel profibrogenic genes regulating collagen production in carbon tetrachloride-induced liver fibrosis.^39^ HSCs with knockdown or knockout of HAS2 show reduced collagen production, HSC migration, and proliferation *in vitro*.^4^,^40^ Also, HAS2 is crucial for HA production, cancer growth in cholangiocarcinoma, and liver metastasis caused by colorectal and pancreatic cancers.^41^,^42^ These findings indicate that HAS2 is the isozyme responsible for HA production in liver fibrosis and liver malignancy; HAS2 and HA are primarily expressed in HSCs; and HAS2 and HA contribute to HSC activation, liver fibrosis, and fibrosis-mediated cancer progression. These studies further suggest that reduced HA clearance and active HA synthesis determine the level of circulating HA in liver fibrosis (Figure 1).

### Molecular mass determines the biological function of HA

HA is synthesized by HAS enzymes and produced as HMM forms (~2×10^6^ Da). HMM-HA is involved in organ development and tissue protection. As such, HMM-HA has been used for cosmetic purposes and for relieving joint pain through local injection. However, once inflammation occurs, HA is fragmented into low molecular mass (LMM)-HA (~1–3×10^5^ Da). HA fragmentation occurs non-enzymatically through reactive oxygen species and enzymatically through hyaluronidases (HYALs).^3^ HYAL1 and HYAL2 play a major role in HA catabolism. HYAL2 is a cell membrane-anchored protein and cleaves HMM-HA to (intermediate) LMM-HA (~20 kDa) in collaboration with CD44. The HMM-HA binding to CD44 induces an acidic extracellular environment by promoting Na^+^/H^+^ exchanger 1(NHE1) activity, allowing HYAL2 to degrade HA to small fragments (~20 kDa). These HA fragments are then taken up to the intracellular acidic endosomal-lysosomal vesicles for further degradation by HYAL1.^43^ Cell migration-inducing protein is also an HYAL that degrades HMM-HA into both intermediate-sized fragments of between 35 and 50 kDa as well as LMM-HA.^44^ PH20, another HYAL, is mainly expressed in sperm cells and is well known for its essential role in fertilization.^3^ Recent studies demonstrate that transmembrane protein 2, a membrane-bound enzyme, can degrade HA in the extracellular environment. Transmembrane protein 2 has intrinsic HYAL activity at neutral pH and is expressed widely in adult mouse tissues, including the lymph nodes and liver.^45^,^46^ Elevated levels of HA in liver fibrosis are attributed to impaired HYAL activity. Interestingly, serum HYAL activity increases in acute hepatitis C, whereas it decreases in chronic hepatitis C.^47^ Transmembrane protein 2 expression decreases in patients with chronic hepatitis B compared with healthy controls.^48^ These reports suggest that HYALs play a role in HA turnover and liver fibrosis. However, the underlying mechanisms of HYAL activity and HA conversion into LMM forms in liver fibrosis still require investigation.

In healthy populations, the molecular size of circulating HA is relatively low (100–300 kDa).^28^,^46^,^49^ In contrast to HMM-HA, LMM-HA is proinflammatory, profibrogenic, and involved in tissue remodeling. Because HA turnover is very short, and the level of circulating HA is low, circulating LMM-HA in a healthy population is not harmful. However, the levels of circulating total and LMM-HA dramatically increase during the progression of liver fibrosis.^4^ Likewise, in fibrotic liver tissues, LMM-HA levels increase.^4^ In current clinical practice, total HA concentrations in blood are measured. LMM-HA is the dominant form of HA in both blood and liver tissues in liver fibrosis.^4^ Given that LMM-HA levels reflect inflammation and HSC activation, measurement of LMM-HA or the ratio of HMM-HA to LMM-HA may be a more sensitive biomarker for liver fibrosis than total HA. Large cohort studies followed by validation studies are required to develop these biomarker recommendations in liver fibrosis. Both LMM-HA and the ratio of HMM-HA to LMM-HA are promising diagnostic and prognostic markers of liver fibrosis and may also reflect the effectiveness of antifibrotic drugs.

### HA receptors and HA-mediated biological functions

HA receptors include CD44, Toll-like receptor 4 (TLR4), TLR2, the receptor for HA-mediated motility expressed protein (RHAMM), and Stabilin-2. CD44 is a classical HA receptor that also binds collagen, fibronectin, and osteopontin. CD44 is widely expressed in immune cells, such as T cells, and is a T-cell activation marker. In acute lipopolysaccharide (LPS)-induced liver injury, HA and CD44 are required for neutrophil recruitment to the injured site.^50^ CD44 blockade inhibits neutrophil recruitment and reduces liver injury, indicating that CD44 promotes LPS-induced liver injury. CD44 is also a marker of cancer stem cells. Normal hepatocytes do not express CD44, whereas transformed HCC cells express CD44 at high levels. Mice with hepatocytes lacking CD44 showed reduced development of HCC, suggesting CD44 is not only cancer stem cells marker but also a functional molecule that contributes to HCC development. In addition to HA binding to CD44 in HCC development, CD44 acts as a co-receptor for EGF receptor and inhibits p53 through AKT and mouse double minute 2. Inhibition of p53 activity results in hepatocyte escape from p53-dependent apoptosis and promotes the reprogramming of hepatocytes into HCC progenitor cells.^51^ CD44 promotes NASH and NASH-associated HCC in mice.^52^,^53^ These reports indicate that CD44 promotes M1 polarization of liver macrophages in NASH and the CD44-HA interaction mediates KC-dependent intrahepatic platelet accumulation in NASH-HCC.

**FIGURE 4:**
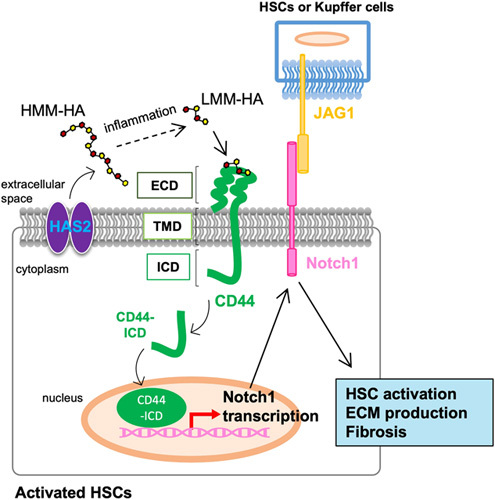
Hyaluronan (HA) promotes HSC activation through CD44-mediated Notch1 transcription. Activation of HSCs and inflammation is the primary driver of liver fibrosis. During the progression of liver fibrosis, hyaluronan synthase 2 (HAS2), the critical hepatic HA synthase, is overexpressed in activated HSCs and produces HA. HA generated as high molecular mass (HMM) forms are converted into low molecular mass (LMM)-HA in the presence of inflammation. LMM-HA binds to and activates CD44, the major HA receptor. CD44 has three domains: an extracellular domain (ECD), a transmembrane domain (TMD), and an intracellular domain (ICD). Activated CD44 proteolytically cleaves CD44-ICD and releases CD44-ICD into the cytoplasm. CD44-ICD translocates to the nucleus and initiates transcription of Notch1. Notch1 receptor interacts with its ligand Jagged-1 (JAG1), derived from nearby liver resident KCs and/or HSCs, to activate profibrogenic Notch1 signaling. HA-mediated CD44-Notch1 activation promotes HSC activation and liver fibrosis.

HSCs express CD44 and TLR4, and HSC activation upregulates the expression of CD44 in liver fibrosis. Both CD44 and TLR4 promote HSC proliferation and invasion induced by HSC-derived HA (Figure 1). However, these receptors regulate distinct gene expression patterns in HSCs. CD44 and TLR4 regulate type I and IV collagens and TIMP1; TLR4 regulates type III collagen; and CD44 regulates type VI collagen.^4^ TLR4 is a crucial receptor for LPS and translocated intestine-derived LPS promotes HSC activation and liver fibrosis.^54^ Similarly, HA-mediated TLR4 activation contributes to liver fibrosis progression.^4^ In HSCs, CD44 contributes to Notch1 activation. HA overexpression and activated CD44 upregulate Notch1 receptor and its ligand Jagged-1 in HSCs.^4^ Briefly, activated CD44 proteolytically cleaves the CD44 intracellular domain, which leads to nuclear translocation of CD44 intracellular domain and upregulation of Notch1 expression (Figure 4).^55^,^56^ CD44-mediated Notch1 activation results in HSC invasion, proliferation, and activation.^4^ Hence, HA promotes fibrogenic, proliferative, and invasive phenotypes of HSCs through the activation of CD44 and TLR4. The different sizes of HA bind CD44 but often lead to other biological actions.^57^ The binding of HMM-HA to CD44 suppresses yes-associated protein (YAP)-mediated cell growth, while the LMM-HA promotes the CD44-YAP-dependent cell growth in vascular smooth muscle cells, fibroblasts, or breast cancer cells. Also, LMM-HA, but not HMM-HA, induces the interaction between CD44 and TLR2/4 in breast tumor cells to promote invasion and cytokine production.^58^,^59^ In HSCs, LMM-HA induces HSC activation through CD44 and TLR4, whereas HMM-HA suppresses or has less effect on HSC activation.^4^ More studies are required to determine molecular size-dependent HA, its receptor binding, and signaling-mediated actions in liver disease.

Less is known about the remaining HA receptors RHAMM and Stabilin-2. A few reports show that HA–RHAMM binding promotes cancer cell motility in HCC and liver metastasis,^60^–^62^ but further studies are necessary to define the mechanistic role of RHAMM in liver disease. Stabilin-2 is a scavenger receptor expressed mainly in LSECs and can bind to HA^63^ (Figure 1). Stabilin-2 knockout mice exhibit high levels of circulating HA, underscoring the contribution of Stabilin-2 expression in LSECs to HA endocytosis and clearance. Interestingly, elevated circulating HA protects against melanoma lung metastasis.^63^ Given that HMM-HA inhibits YAP-mediated tumor growth through CD44 and that LMM-HA enhances YAP-mediated tumor growth by binding to CD44,^59^ increased circulating HA after Stabilin-2 ablation may be due to the tumor-suppressive properties of HMM-HA. However, this hypothesis requires additional investigation. Collectively, HA receptors play major roles in disease-regulating signaling pathways in immune cells, cancer cells, HSCs, and LSECs.

### Regulatory mechanisms of HAS2 expression in HSCs

**FIGURE 5:**
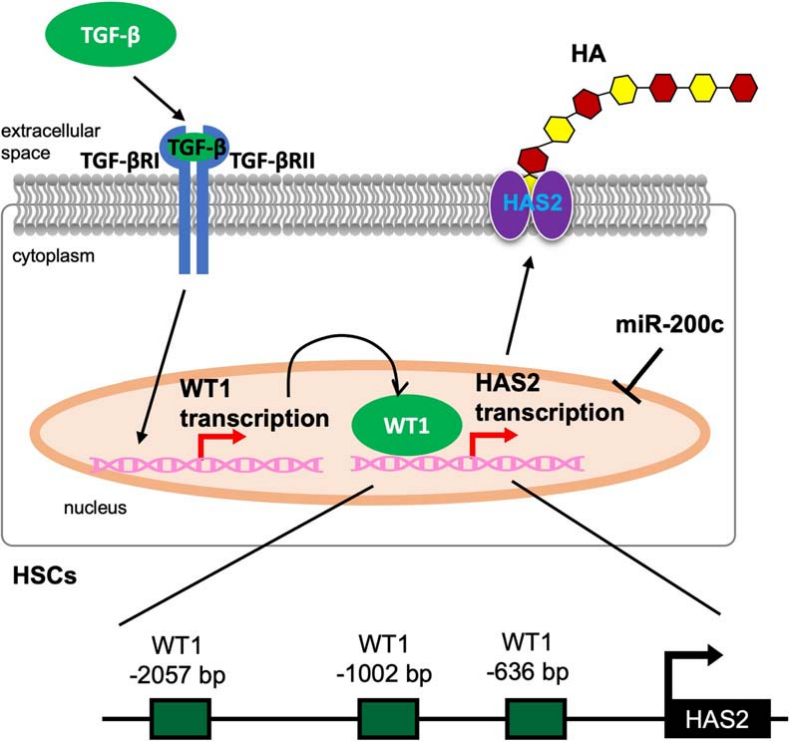
Regulation of hyaluronan synthase 2 (HAS2) in HSCs. In HSCs, TGF-β initiates intracellular signaling by binding and activating 2 TGF-β receptor I (TGF-βRI) and II (TGF-βRII) and activated TGF-β receptor signaling induces Wilms tumor 1 (WT1) expression. Subsequently, WT1 binds to the promoter region of HAS2. All 3 WT1 binding sites in the HAS2 promoter (–2057, –1002, and –636 bp from the transcription start site) are required for activation. HAS2 is also post-transcriptionally regulated by miR-200c binding to the 3’-UTR of HAS2 mRNA.

HSCs are responsible for HAS2 expression and HA synthesis in liver fibrosis.^4^ Quiescent HSCs do not express HAS2 or produce HA.^4^ Upon HSC activation, HAS2 expression and HA production are dramatically upregulated.^4^ Like ECM-producing lung fibroblasts, TGF-β is a potent inducer of HSC transdifferentiation to myofibroblasts and HAS2 overexpression. The HAS2 promoter does not contain conserved binding sites for Smad transcription factors, which are activated by TGF-β, suggesting an alternative transcriptional mechanism for the upregulation of HAS2 in response to TGF-β.^24^ T-box 4, a transcription factor upregulated by TGF-β in lung fibroblasts,^64^ is not induced by TGF-β in HSCs (unpublished observations, Y.M. Yang and E. Seki). Instead, the HAS2 promoter contains 3 putative binding sites for Wilms tumor 1 (WT1) (Figure 5).^4^ WT1 is a transcription factor expressed in cells originating from the mesoderm and is also expressed in HSCs.^65^ TGF-β induces the upregulation of both WT1 and HAS2, and WT1 knockdown abolishes TGF-β-induced HAS2 induction, indicating that TGF-β-mediated WT1 transcriptionally regulates HAS2 expression in HSCs.^4^ In contrast, another study demonstrated that WT1 negatively regulates HSC activation in carbon tetrachloride–induced liver fibrosis.^66^ Although different experimental approaches are used (eg, other Cre mice selected to target HSCs and different liver fibrosis models), future studies are necessary to unveil the precise mechanisms of HSC activation related to WT1 and HAS2.

HAS2 expression is also regulated epigenetically.^40^ For example, an epigenetic regulator methyl-CpG binding protein 2 regulates the expression of profibrogenic genes through phosphorylation at serine 80 in HSCs. HAS2 is one of the profibrogenic genes regulated by methyl-CpG binding protein 2. A post-transcriptional regulation of HAS2 was also reported. miR-200c is downregulated by liver fibrosis, binds to the 3’-UTR of HAS2, and post-transcriptionally regulates HAS2 expression in HSCs (Figure 5).^67^ Hedgehog (Hh) signaling is another important pathway in HSC activation and liver fibrosis. In Hh signaling, the interaction of sonic Hedgehog ligand and the cell surface receptor Patched releases and activates Smoothened, leading to nuclear localization of glioblastoma family transcription factors (Glis) that regulate the expression of cell-specific target genes. Although not reported in the liver, HAS2 is a direct target of Gli transcriptional regulation during early mouse limb development.^68^ Gli3 binds to the HAS2 promoter region, and HAS2 is required to establish joint patterning within digits as an important downstream effector of sonic Hedgehog ligand signaling. Although there is no direct evidence for Hh-mediated HAS2 regulation in the liver, a few reports suggest Hh signaling interacts with HA production in NAFLD.^69^ Therefore, further studies are required to define the connection between HAS2 and Hh signaling. Many questions still remain regarding the regulatory mechanism of HAS2 in the liver because the importance of HAS2 in the liver has only recently attracted interest.

### Targeting HA as a therapeutic strategy for liver disease

**FIGURE 6:**
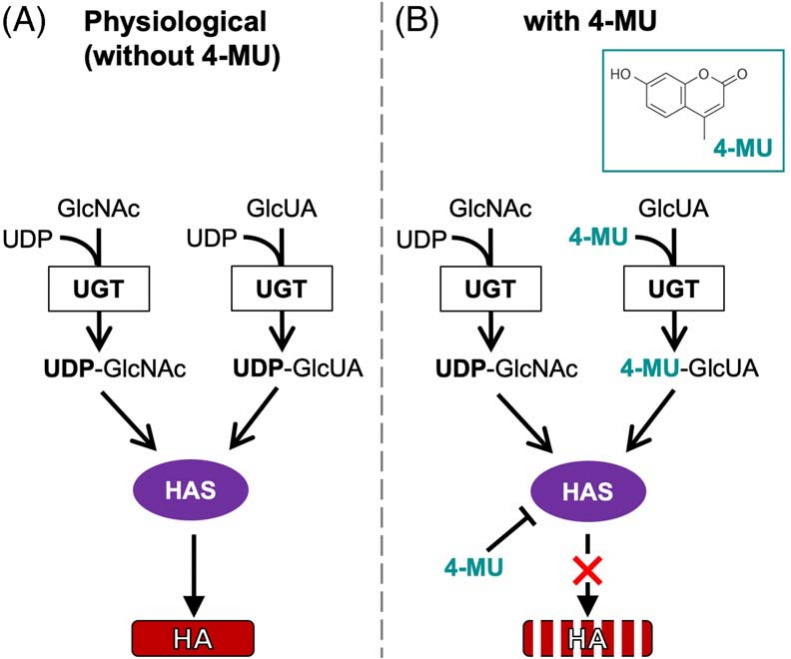
Postulated mechanism of the inhibitory effect of 4-methylumbillifelone (4-MU) on hyaluronan (HA) synthesis. HA synthesis is inhibited by the small molecule inhibitor 4-MU, a coumarin derivative. Functionally, 4-MU is a competitive substrate for uridine diphosphate (UDP)-glucuronosyltransferase (UGT), an enzyme involved in HA synthesis. (A) In the absence of 4-MU, HA is produced by hyaluronan synthase (HAS) from UDP-N-acetyl-glucosamine (GlcNAc) and UDP-glucuronic acid (GlcUA). These substrates are generated by the transfer of a UDP residue to Glc-NAc or Glc-UA via UGT. Thus, the availability of UDP-GlcNAc and UDP-GlcUA limits HA synthesis. (B) In the presence of 4-MU, 4-MU binds to GlcUA and depletes the pool of UDP-GlcUA, which is required for HA synthesis. Thus, 4-MU restricts the availability of an essential substrate for HA synthesis.

There are 2 approaches to target HA in liver disease: inhibition of HA synthesis and targeted degradation of HA. The coumarin derivative (7-hydroxy-4-methylcoumarin), 4-methylumbillifelone (4-MU), also known as hymecromone, is used to treat biliary spasm in Asia and Europe and inhibit HA synthesis. Mechanistically, 4-MU competes with UDP-GlcUA as a substrate for UDP-glucuronosyltransferase, effectively inhibiting the synthesis of HA (Figure 6). 4-MU also suppresses HAS2 and HAS3 transcription^70^ to inhibit HA synthesis. In HSCs, 4-MU treatment inhibits HA production and collagen expression and suppresses liver fibrosis induced by cholestasis, carbon tetrachloride, and NASH in mice.^4^,^71^,^72^ Because HA contributes to HCC development, 4-MU treatment inhibits HCC growth in mice.^73^ Although previous reports suggest that a high dose of 4-MU is required due to its rapid clearance and low systemic bioavailability (<3%), a recent study revealed that 4-methylumbelliferyl glucuronide (4-MUG), the main metabolite of 4-MU, also has bioactivity. 4-MUG inhibits HA synthesis and further converts back into 4-MU in vivo.^74^ This suggests that the bioavailability of 4-MU is higher than what we previously thought. Hence, further pharmacokinetics and pharmacodynamics studies for 4-MU and 4-MUG are still needed.^75^ Several clinical trials using 4-MU were conducted and reported it is a safe and well-tolerated oral medication that decreases HA levels in the serum and sputum of healthy subjects.^70^,^76^ This suggests its efficacy in pulmonary disease. Future studies are required to reveal its efficacy in liver disease, including liver fibrosis. At the same time, we must be cautious of the systemic effect of 4-MU outside the liver. In addition to 4-MU, etoxazole, a chitin synthesis inhibitor, was reported as an HA inhibitor. Etoxazole reduces HA production and prevents collagen accumulation in carbon tetrachloride-induced liver fibrosis.^77^ Importantly, etoxazole has a half-maximal inhibitory concentration (IC50) of HA deposition in a lower micromolar range (4.21±3.82 μΜ) than 4-MU (IC50=8.68±1.6 μΜ), suggesting that etoxazole has therapeutic potential to inhibit HA at a lower dose than 4-MU.

Degradation of HA by HYALs is an alternative approach to reducing local HA abundance. In colorectal cancer liver metastasis, chemoresistance, and HA deposition are often observed after anti-VEGF therapy. Accumulation of HA in cancer is associated with increased tumor stiffness and prevents chemotherapeutics from reaching cancer cells. In a preclinical mouse model, PEGylated HYAL PH20 administration degrades accumulated HA in liver metastasis, allowing anti-cancer chemotherapeutics to reach cancer cells and resulting in increased efficacy of anti-VEGF plus chemotherapy.^78^ The recent phase III clinical trial reported that PEGylated HYAL PH20 did not improve overall and progression-free survivals in pancreatic cancer patients with nab-paclitaxel/gemcitabine treatment.^79^ However, approaches to degrade HA may still be applied to treat liver malignancy, including HCC with cirrhosis, which often has strong fibrous capsules surrounding tumors.

As mentioned previously, the molecular mass of HA impacts biological function. HA35, the 35 kDa form of HA, has anti-inflammatory effects, distinct from most proinflammatory LMM-HA. HA35 inhibits TLR4 signaling by decreasing importin α5 and increasing Tollip, which results in the inhibition of alcohol-induced liver injury in rodents.^80^,^81^ Thus, HA35 may be a treatment option for TLR4-mediated liver disease progression. However, the stability of HA35 must be carefully considered because degradation of HA35 to smaller molecular mass could be pro-inflammatory and difficult to control.

Given that cancer cells and cancer stem cells overexpress CD44, HA-based drug, and nucleotide delivery systems may be an effective treatment strategy. HA-paclitaxel conjugate, doxorubicin, and gemcitabine conjugated to an HA backbone, and lipid nanoparticles containing paclitaxel and targeted with HA are currently tested for the treatment of bladder, ovarian, and breast cancer as well as melanoma.^82^ HA-based nanoparticles can deliver specific microRNAs (eg, miR-29, miR-125, miR-155) to macrophages, polarizing them from pro-cancer M2 to anti-cancer M1 states.^82^ Because liver cancer cells, liver macrophages, and activated HSCs express CD44 at high levels, HA-based delivery systems may be an effective treatment for liver cancer, ALD, NASH, and liver fibrosis. However, HA components may stimulate proinflammatory, profibrotic, and procancer CD44 signaling, limiting the application of this approach. Additional studies are necessary to investigate the therapeutic potential of HA-based CD44 targeting systems in liver disease.

Obesity is a systemic disease that affects NASH progression and insulin resistance. Obesity can be treated using 4-MU, which acts on brown adipose tissues. A recent report demonstrates that 4-MU treatment inhibits diet-induced weight gain and attenuates insulin resistance independent of HA.^83^ Briefly, 4-MU treatment changes the metabolic activity of brown adipose tissues. Sugar precursors (UDP-GlcUA and UDP-GlcUAc) are no longer used for HA synthesis, leading to increased glycolysis and mitochondrial respiration in brown adipose tissues and mitigation of obesity and diabetes. This alternative pathway is suggested as the mechanism by which HA synthesis affects disease progression.^83^

### Concluding remarks

In hepatology research and clinical practice, HA is a classic biomarker of cirrhosis. Although disruption of HA clearance is recognized for its role in cirrhosis, HA hypersynthesis has not been thoroughly investigated. Additional studies are still needed to elucidate regulatory mechanisms and downstream effectors of HA. A recent study established a connection between enhanced HA synthesis and HAS2 overexpression in activated HSCs during liver fibrosis. The bidirectional regulation between HA and HSCs is crucial for HSC activation, ECM deposition, and fibrosis. HSC-derived HA also affects surrounding liver cells, including hepatocytes, KCs, and LSECs, further facilitating HSC activation and fibrosis. Although a recent intriguing report shows that inhibition of HA synthesis causes previously underappreciated HA-independent biological mechanisms,^83^ targeting HA synthesis can be an attractive treatment approach for fibrotic liver disease as well as liver malignancy. Repurposing 4-MU or additional development of new 4-MU derivatives or small molecules should also be considered. The effects of different molecular mass HA are validated. Although further studies of the mechanisms of HA catabolism are desired, the detection of different molecular mass HA may become a valuable diagnostic tool. New diagnostic tools will also help to validate the effectiveness of novel antifibrotic drugs. Targeting HA is a valuable approach for diagnosing and treating liver fibrosis.
